# 

**Published:** 1886-01-20

**Authors:** 


### TABLE OF CONTENTS.

ORIGINAL AND SELECTED ARTICLES.

Gun-Shot Wound Through the Bladder, by J. McF. Gaston, M. D., Professor of the Principles and Practice of Surgery in the Southern Medical College, Atlanta, Georgia.................... 1
Practical Dots, by T. L. Lallerstedt, M.D., of Ga. 2
A Plea for Plaster of Paris in the Treatment of Fracture, by L. Edwards Borcheim, M. D., Atlanta, Ga........................................................ 5
Case of Abdominal Tumor, with Autopsy, Presented to the Atlanta Society of Medicine by Dr. W. P. Nicolson. Reported for the Southern Medical Record........................................... 6
Exophthalmic Goitre, or Graves' Disease. by J. W. Thompson, M.D., of St. Paul, Minn...........11
Punctured Wound of Heart, by Chas. Chassaignac, M. D., of Louisiana...............................15
Acid Phosphates; How to Blister Quickly; A New Anti-epileptic and Nervine; Application for Neuralgia..................................................16

ABSTRACTS AND GLEANINGS.

Prevention of Septicæmia; Alcoholic Stupor...17
Opium Poisoning; Lengthening of the Child-bearing Period in American Women...............18
Dr. Ultman's Treatment of Functional Impotence; Transfixion of the Scrotum...................19
Cutaneous Hæmorrhage by Auto-Suggestion; A New Remedy for Sea-sickness...................... 20
Cocaine Intoxication; Diabetes Successfully Treated with Boracic Acid....................................21
Tedious Labor..........................................................22
Gun Shot Wounds..................................................24
Oil of Eucalyptus and Oil of Turpentine in Membraneous Croup; Migraine—What is its Cause.....................................................................25
Treatment of the Opium Habit; The Treatment of Frost-Bitten Fingers and Toes; for Spinal Tenderness .................................................................26
The Action of Antipyrine upon the Croupous Pneumonia of Children; Treatment of Fainting; Antipyrine...................................................27
Asthma as Related to Skin Diseases; Alcohol as a Hypnotic in Fevers; Glyclosuria...................28
External Use of Chloral Hydrate; Treatment of Acute Rheumatism........................................29

SCIENTIFIC ITEMS.

Inoculation in Yellow Fever..................................31
Resisting Electric Shocks; The "Big Woods" of Minnesota. ......................................................32

PRACTICAL NOTES AND FORMULÆ.

Sciatica; Acute Pleurisy; Dyspepsia; Chilblain.33
Wine of Pepsin; For Vomiting of Pregnancy; Chilblains...........................................................34

EDITORIALS AND MISCELLANEOUS.

Collaborators; Squibbs' Epidermis; Advertisements..........................................................35
Yellow Fever Inoculation; Medical Society of the State of Tennessee; Fine Display of Chemicals, etc; Thermometers..................................36
Ivy-Street Hospital; Protection from Yellow Fever by Inoculation............................................37
The Metric System of Weights and Measures; Books and Pamphlets Received.........................38
Receipted; Special Notices....................................40

